# Evaluating the Construct Validity of the Charité Alarm Fatigue Questionnaire using Confirmatory Factor Analysis

**DOI:** 10.2196/57658

**Published:** 2024-08-08

**Authors:** Maximilian Markus Wunderlich, Henning Krampe, Kristina Fuest, Dominik Leicht, Moriz Benedikt Probst, Julian Runge, Sebastian Schmid, Claudia Spies, Björn Weiß, Felix Balzer, Akira-Sebastian Poncette, CAFQa Study Group Germany, Mirza Aghamov

**Affiliations:** 1 Institute of Medical Informatics, Charité–Universitätsmedizin Berlin, Corporate Member of Freie Universität Berlin and Humboldt-Universität zu Berlin, Charitéplatz 1, Berlin, 10117, Germany, 49 30 450 581018; 2 Department of Anesthesiology and Intensive Care Medicine CVK/CCM, Charité–Universitätsmedizin Berlin, Corporate Member of Freie Universität Berlin and Humboldt-Universität zu Berlin, Berlin, Germany; 3 Department of Anaesthesiology & Intensive Care Medicine, Klinikum Rechts der Isar, School of Medicine, Technical University Munich, Munich, Germany; 4 Department for Anaesthesiology, Intensive Care Medicine and Pain Therapy, Justus Liebig University, Giessen, Germany; 5 Department for Anaesthesiology, Intensive Care Medicine and Pain Therapy, Vivantes Klinikum im Friedrichshain, Berlin, Germany; 6 Department for Anesthesiology, Surgical Intensive Care, Pain and Palliative Medicine, Marien Hospital Herne–Universitätsklinikum der Ruhr-Universität Bochum, Herne, Germany; 7 Department of Anesthesiology and Intensive Care Medicine, University Hospital Ulm, Ulm University, Ulm, Germany

**Keywords:** patient monitoring, intensive care unit, alarm, alarms, validity, validation, safety, intensive, care, alarm fatigue, alarm management, patient safety, ICU, alarm system, alarm system quality, medical devices, clinical alarms, questionnaire, questionnaires, warning, factor analysis

## Abstract

**Background:**

The Charité Alarm Fatigue Questionnaire (CAFQa) is a 9-item questionnaire that aims to standardize how alarm fatigue in nurses and physicians is measured. We previously hypothesized that it has 2 correlated scales, one on the psychosomatic effects of alarm fatigue and the other on staff’s coping strategies in working with alarms.

**Objective:**

We aimed to validate the hypothesized structure of the CAFQa and thus underpin the instrument’s construct validity.

**Methods:**

We conducted 2 independent studies with nurses and physicians from intensive care units in Germany (study 1: n=265; study 2: n=1212). Responses to the questionnaire were analyzed using confirmatory factor analysis with the unweighted least-squares algorithm based on polychoric covariances. Convergent validity was assessed by participants’ estimation of their own alarm fatigue and exposure to false alarms as a percentage.

**Results:**

In both studies, the *χ*^2^ test reached statistical significance (study 1: *χ*^2^_26_=44.9; *P*=.01; study 2: *χ*^2^_26_=92.4; *P*<.001). Other fit indices suggested a good model fit (in both studies: root mean square error of approximation <0.05, standardized root mean squared residual <0.08, relative noncentrality index >0.95, Tucker-Lewis index >0.95, and comparative fit index >0.995). Participants’ mean scores correlated moderately with self-reported alarm fatigue (study 1: *r*=0.45; study 2: *r*=0.53) and weakly with self-perceived exposure to false alarms (study 1: *r*=0.3; study 2: *r*=0.33).

**Conclusions:**

The questionnaire measures the construct of alarm fatigue as proposed in our previous study. Researchers and clinicians can rely on the CAFQa to measure the alarm fatigue of nurses and physicians.

## Introduction

### Background

Alarm fatigue is a phenomenon where health care workers in intensive care units (ICUs) become desensitized to alarms of medical devices [1]. It can make ICU staff feel stressed, and it is a substantial risk to patient safety, as it can lead to alarms being missed or acknowledged with delay [2]. When implementing interventions or IT solutions [3] that try to remedy alarm fatigue, clinicians and clinical alarm researchers need a reliable way to assess whether they were successful. However, they have not yet agreed on a standardized way of measuring alarm fatigue [4,5], even though it was recognized more than 2 decades ago [6].

Solely analyzing an ICU’s alarm log data cannot serve as a measure of staff’s alarm fatigue. While it is a valuable method for designing alarm management interventions [7], there is no clear association between the number of alarms on an ICU and staff’s subjective alarm fatigue. For example, simply focusing on the number of alarms disregards their temporal distribution (eg, it might fatigue staff more if alarms came in random bursts than if they were evenly spaced out [8]). This could be one of the reasons why in their intervention study, Sowan et al [9] did not find that staff’s alarm fatigue improved despite having managed to significantly reduce the number of alarms on their ICU.

Therefore, we recently developed the Charité Alarm Fatigue Questionnaire (CAFQa), which is a 9-item questionnaire that measures alarm fatigue in nurses and physicians [10]. Using exploratory factor analysis, we identified 2 correlated factors: one revolving around the psychophysiological effects of alarms (eg, headaches and feelings of distraction), and one revolving around ICU staff’s alarm management strategies (eg, customization of alarm limits). We named the former the “alarm stress scale” and the latter the “alarm coping scale.” The alarm coping scale consists of items that are reversely scored. Hence, a high score on either scale is indicative of alarm fatigue.

When developing a new questionnaire, it is essential to establish construct validity, that is, whether the questionnaire truly measures what it attempts to measure. One way to test an instrument’s construct validity is to administer it to a different sample and test whether the originally proposed factor structure reemerges using confirmatory factor analysis [11,12] (for a recent example see Canivez et al [13]).

### Aim

We aim to validate the exploratively derived factor structure of the CAFQa and thus underpin the instrument’s construct validity.

## Methods

### Ethical Considerations

The ethical approval for this study was granted by the ethics committee of the Charité–Universitätsmedizin Berlin (EA4/218/20) and, if required, confirmed by the local ethics committee at the participating hospital. This study was conducted in compliance with the relevant guidelines and regulations. All participants voluntarily agreed to take part after being fully informed about the study. In study 1, as a reward for completing the questionnaire, we offered participants the chance to enter a draw where they could win a €50 (US $53) voucher for online shopping. Participants were asked to consent to have their data collected, analyzed, and stored anonymously.

### Participants

In both studies, we included nurses, physicians, and nurses in training, while excluding other professions and non-ICU staff.

#### Study 1

We recruited participants from 9 ICUs of 5 large German hospitals. The questionnaire was administered on the web using REDCap (Research Electronic Data Capture; Vanderbilt University) between October 2021 and July 2022.

#### Study 2

Using a mailing list, we invited all members of the German Society of Anaesthesiology and Intensive Care Medicine [14] to fill out the web-based questionnaire (again using REDCap) between March 2023 and July 2023.

### Questionnaire

The questionnaire used in both studies was identical and consisted of all 9 items from the CAFQa [10] and 5 general questions about the alarm situation in participants’ ICUs. These general questions were not part of the analysis for this report. All 14 items were pseudorandomly arranged and required responses on a Likert scale ranging from −2 (indicating “I do not agree at all”) to 2 (indicating “I very much agree”). Items with negative valences were reverse scored. Demographic items asked participants about their average number of workdays in an intensive care or monitoring area, their number of years and months of ICU experience, their workplace (campus and unit), and their profession. We made small adjustments to the original wording of 2 items (items 8 and 9) to improve readability: In item 8 we used “situation” instead of “urgency.” In item 9 we used the phrase “clinical pictures” instead of “clinical symptoms.”

### Statistical Analysis

All analyses were conducted in R (version 4.2.1; R Foundation for Statistical Computing) using the following packages: *Tidyverse* [15], *reshape2* [16], *psych*, *semPlot* [17], and *lavaan* [18]. For study 1, we pooled the data from the participating hospitals.

#### Missing Data

In accordance with Heymans and Eekhout [19], we used predictive mean matching via the *mice* package [20] to impute missing data that were assumed to be missing at random (MAR). We did not impute questionnaires that were either completely empty or terminated prematurely (presumably due to survey fatigue), as the assumption of MAR was not met in these cases. We assumed that survey fatigue occurred if a participant failed to respond to at least the final 20% of the questionnaire (ie, the last 3 or more of the 9 items of the CAFQa plus the 5 general questions). In total, 0.3% of the data were MAR.

#### Testing Assumptions of Confirmatory Factor Analysis

In both studies, the results of the Mardia test indicated that the multivariate skew did not come from a normal distribution with *P*<.001. Outliers were identified using Mahalanobis distances, with none being detected in study 1 and 4 being detected in study 2 (for both studies: *χ*^2^_9_ cutoff=27.9; *P*<.001). Visual inspection of the data from all 4 cases revealed no unusual response patterns. Given the large sample size, we decided not to remove any outliers. The Kaiser-Meyer-Olkin statistic [21] in study 1 was 0.76, and in study 2 it was 0.8. In both studies, the Bartlett test of sphericity [22] rejected the null hypothesis that the correlation matrix was an identity matrix (study 1: *χ*^2^_36_=438.3; *P*<.001; study 2: *χ*^2^_36=_2495.4). There was no evidence of multicollinearity in either study as the determinant of both *R* matrices was greater than 0.00001 [23] and no correlations were greater than |0.7|. Overall, these results suggest that the data of both studies were suitable for factor analysis.

#### Confirmatory Factor Analysis

For both studies, we specified the model in line with our previous findings [10], with 2 correlated latent factors, labeled “alarm stress” and “alarm coping.” Items 1‐5 were assigned to “alarm stress.” Items 5‐9 were assigned to “alarm coping.” Since all CAFQa items are ordered categorical variables (due to being measured on a 5-point Likert scale) and because the Mardia tests indicated that the multivariate skew of both studies did not come from a normal distribution, we used the unweighted least-squares (ULS) algorithm based on polychoric covariances for estimating factor loadings [24-26]. We assessed the goodness-of-fit of the model using *χ*^2^, and the following fit indices in line with the cutoff criteria defined by Hu and Bentler [27]: root mean square error of approximation (RMSEA), relative noncentrality index (RNI), Tucker-Lewis index (TLI), standardized root mean squared residual (SRMR), and comparative fit index (CFI).

#### Convergent Validity

At the end of the questionnaire in both studies, we provided participants with a brief description of alarm fatigue and asked them to estimate their personal alarm fatigue as a percentage (0% indicating no alarm fatigue and 100% indicating extreme alarm fatigue). We also asked participants to provide their perceived rate of false alarms in their ICU as a percentage (0% indicating no false alarms, 100% indicating no true alarms). To measure convergent validity, we correlated the participants’ mean scores on the questionnaire with the self-provided alarm fatigue and false alarm rate estimations (in total and per factor).

#### Internal Consistency

As a measure of internal consistency, we report Cronbach coefficient α, the McDonald coefficient ω [28], and the mean interitem correlation for both factors.

## Results

### Participants

#### Study 1

We received 363 submissions. Among these, 23 came from participants who did not consent to have their data analyzed, 67 questionnaires were empty, and 8 showed signs of survey fatigue. Therefore, the sample size for this study was 265. The number of participants was roughly similar for each hospital (Giessen: n=43; Herne: n=50; Munich: n=64; Ulm: n=57; Vivantes: n=51). Most participants were nurses (n=150, 56.6%) and 35.8% (n=95) were physicians. A few participants (n=9, 3.4%) were supporting nurses, nurses in training, medical students, or interns, while 4.2% (n=11) did not state their profession.

#### Study 2

Of the 1564 submissions we received, 69 came from participants who refused to consent to have their data processed and 223 were empty questionnaires. We suspected survey fatigue in 60 cases. Hence, the sample size of study 2 was 1212. Contrary to study 1, more participants were physicians (n=1002, 82.7%) than nurses (n=186, 15.3%). Again, the group of supporting nurses, nurses in training, medical students, and interns was a minority (n=6, 0.5%). Among the participants, 1.5% (n=18) did not state their profession.

### Confirmatory Factor Analysis

Descriptive statistics of both studies are presented in Table 1 for each item.

**Table 1.** Descriptive statistics for each item and the pattern coefficients found in the confirmatory factor analysis of the 2-factor model in both studies. All loadings were statistically significant at *P*<.001.

Item	Description	Study 1					Study 2				
		Factor 1 (95% CI)	Factor 2 (95% CI)	Mean (SD)	Kurtosis	Skew	Factor 1 (95% CI)	Factor 2 (95% CI)	Mean (SD)	Kurtosis	Skew
1	With too many alarms on my ward, my work performance, and motivation decrease.	0.730 (0.643‐0.818)	—^a^	0.47 (1.01)	–0.62	–0.3	0.677 (0.636‐0.717)	—	0.51 (1.08)	–0.63	–0.37
2	Too many alarms trigger physical symptoms for me, e.g., nervousness, headaches, and sleep disturbances.	0.706 (0.612‐0.800)	—	0.23 (1.26)	–1.13	–0.16	0.694 (0.653‐0.735)	—	0.22 (1.20)	–1.00	–0.14
3	Alarms reduce my concentration and attention.	0.725 (0.635‐0.814)	—	0.43 (1.07)	–0.91	–0.21	0.813 (0.779‐0.846)	—	0.63 (1.03)	–0.59	–0.39
4	My or neighboring patients’ alarms or crisis alarms frequently interrupt my workflow.	0.432 (0.318‐0.547)	—	0.87 (0.83)	–0.41	–0.39	0.519 (0.469‐0.570)	—	0.70 (0.87)	–0.28	–0.37
5	There are situations when alarms confuse me.	0.488 (0.384‐0.593)	—	0.08 (1.09)	–0.73	–0.09	0.634 (0.592‐0.676)	—	0.19 (1.07)	–0.85	–0.04
6	In my ward, procedural instruction on how to deal with alarms is regularly updated and shared with all staff.^b^	—	0.434 (0.270‐0.598)	0.77 (1.24)	–0.68	–0.7	—	0.449 (0.375‐0.523	1.10 (1.02)	0.44	–1.06
7	Responsible personnel respond quickly and appropriately to alarms.^b^	—	0.587 (0.424‐0.750)	-0.32 (0.82)	–0.12	–0.12	—	0.639 (0.567‐0.711	–0.39 (0.90)	–0.05	0.19
8	The acoustic and visual monitor alarms used on my ward floor and in my nurses’ station allow me to assign the patient, the device, and the situation clearly.^b^	—	0.349 (0.182‐0.517)	-0.46 (1.06)	–0.52	0.36	—	0.428 (0.359‐0.498	–0.36 (1.15)	–0.66	0.32
9	Alarm limits are regularly adjusted based on patients’ clinical pictures (e.g., blood pressure limits for conditions after bypass surgery).^b^	—	0.581 (0.428‐0.734)	-0.38 (0.93)	–0.24	0.26	—	0.575 (0.508‐0.641	–0.35 (0.94)	–0.12	0.40

a Not applicable.

b Item with a negative valence that is reversely scored.

#### Study 1

While the *χ*^2^ test was significant at α=.05 with *χ*^2^_26_=44.9 *(P*=.01), indicating that the model did not fit the data, all fit indices suggested a good model fit: RMSEA=0.03, SRMR=0.052, RNI=0.989, TLI=0.985, and CFI=0.989. All items loaded onto their hypothesized factors as expected, with factor loadings that were statistically significant at *P*<.001, ranging from 0.35 to 0.73. The factors were moderately correlated at 0.4 (95% CI 0.21‐0.59; *P*<.001).

#### Study 2

**Figure 1.**
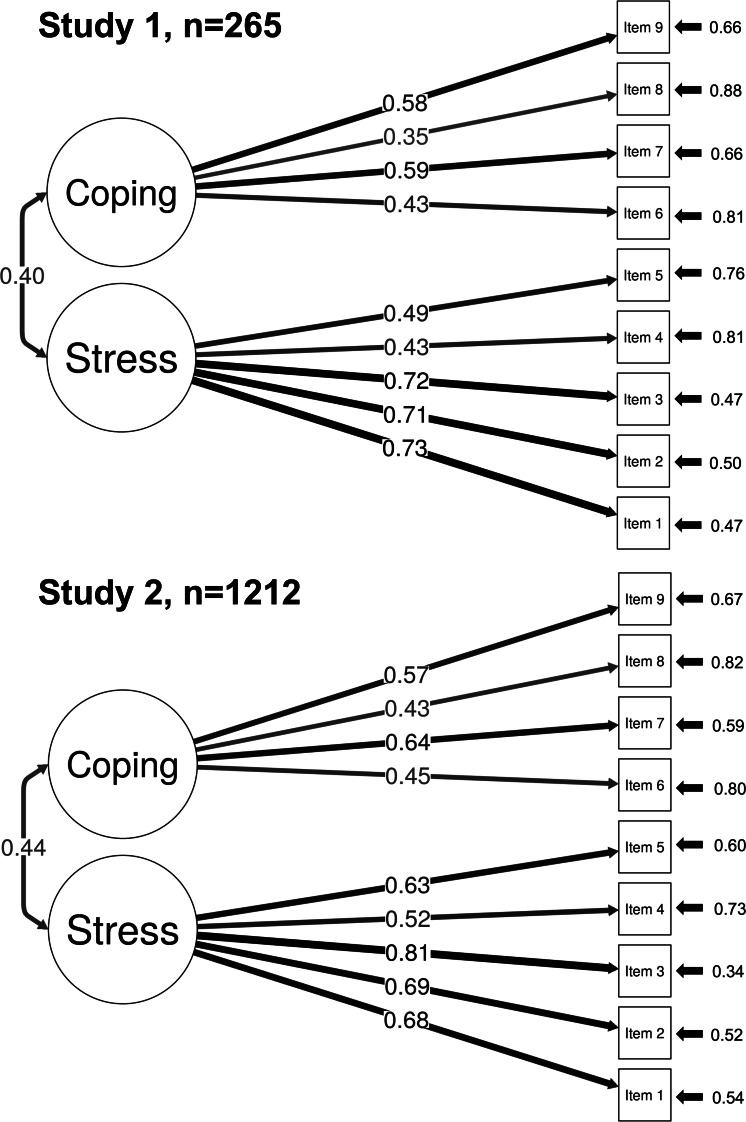
Diagram of the final model from each study. Factors are shown in circles (factor 1: alarm stress; factor 2: alarm coping), and items 1‐9 in squares. In both studies, the variance of the factors was constrained to 1. The arrows connecting the factors with the items are the factor loadings and arrows pointing toward the items show the residuals. The arrows between the factors show their correlation.

As in study 1, the *χ*^2^ test was significant (*χ*^2^_26_=92.4; *P*<.001), indicating that the model did not fit the data, while the fit indices showed a good model fit: RMSEA=0.046, SRMR=0.041, RNI=0.982, TLI=0.975, and CFI=0.982. Again, all items loaded onto their hypothesized factors as expected, with factor loadings that were statistically significant at *P*<.001, ranging from 0.43 to 0.81. The factors were moderately correlated at 0.44 (95% CI 0.36‐0.51; *P*<.001) (Figure 1).

### Convergent Validity

In study 1, the participants’ mean scores on the questionnaire correlated moderately with self-reported alarm fatigue (*r*_242_=0.45, 95% CI 0.34-0.54; *P<.*001) and weakly with the perceived percentage of false alarms (*r*_247_=0.3, 95% CI 0.18 to –0.41; *P*<.001). Similar patterns were observed in study 2 (Table 2 provides full details).

**Table 2.** Correlation coefficients, *P* values, and CIs used to investigate the convergent validity in each study.

Study and correlation measure		*r* (*df*; 95% CI)	*P* value
**Study 1**			
	MS-SRAF^a^^b^	0.45 (242; 0.34‐0.54)	<.001
	F1S-SRAF^c^	0.42 (242; 0.31‐0.52)	<.001
	F2S-SRAF^d^	0.29 (242; 0.17‐0.4)	<.001
	MS-PPFA^e^	0.30 (247; 0.18‐0.41)	<.001
	F1S-PPFA	0.20 (247; 0.08‐0.32)	.002
	F2S-PPFA	0.29 (247; 0.17‐0.4)	<.001
**Study 2**			
	MS-SRAF	0.53 (1180; 0.49‐0.57)	<.001
	F1S-SRAF	0.49 (1180; 0.45‐0.53)	<.001
	F2S-SRAF	0.34 (1180; 0.29‐0.39)	<.001
	MS-PPFA	0.33 (1182; 0.28‐0.38)	<.001
	F1S-PPFA	0.26 (1182; 0.21‐0.32)	<.001
	F2S-PPFA	0.28 (1182; 0.23‐0.33)	<.001

a MS: mean score on the questionnaire.

b SRAF: self-reported alarm fatigue.

c F1S: scores on factor 1.

d F2S: scores on factor 2.

e PPFA: perceived percentage of false alarms.

### Internal Consistency

In study 1, the Cronbach α of factor 1 was 0.72, and it was 0.49 for factor 2. Cronbach α across factors was 0.67. The mean interitem correlation on factor 1 was 0.38, and it was 0.23 on factor 2. The McDonald coefficient ω for factor 1 was 0.77, and for factor 2 it was 0.55. The overall coefficient ω for the assessment was 0.8.

Results were similar in study 2: the Cronbach α was 0.77 for factor 1 and 0.55 for factor 2. Cronbach α across factors was 0.72. The mean interitem correlation was 0.44 on factor 1 and 0.27 on factor 2. The McDonald coefficient ω was 0.8 for factor 1 and 0.59 for factor 2. The overall coefficient ω for the assessment was 0.85.

## Discussion

We aimed to underpin the construct validity of the CAFQa by submitting the exploratively derived factor structure from our previous study to confirmatory factor analysis in 2 independent studies. While the *χ*^2^ test rejected the model in both studies, all fit indices indicated a good model fit. The factor loadings ranged from 0.35 to 0.73 in study 1 and from 0.43 to 0.81 in study 2; all were statistically significant. Overall, these results support the hypothesized factor structure. The questionnaire seems to measure the construct of alarm fatigue as proposed in our previous work [10].

The *χ*^2^ test is known to be sensitive to large sample sizes [29], which might explain its statistical significance. We did not modify the model because all fit indices indicated a good fit and because model modifications, no matter how small or plausible, can make a model less generalizable.

In both studies, the first factor, that is, the alarm stress scale, and the overall questionnaire were internally consistent. However, the second factor, that is, the alarm coping scale, seems to have issues with its internal consistency. Here, Cronbach α and McDonald ω were 0.49 and 0.55 in study 1, respectively, and 0.55 and 0.59 in study 2, respectively. A similar pattern can be found in our previous study, where the Cronbach α of factor 2 was 0.57 [10]. An internally consistent questionnaire is desirable. However, it can also mean that items are very similar. It was our ambition to create a questionnaire that is brief while measuring the many facets of alarm fatigue. Future studies using the CAFQa should routinely assess the internal consistency of both factors. If the second factor continues to show medium internal consistency, research should be done on how it can be improved (eg, by adding additional items, which would come at the cost of a longer questionnaire).

Because no other measures of alarm fatigue exist that would allow us to estimate the CAFQa’s convergent validity, we asked participants to rate their own alarm fatigue as well as the rate of false alarms they perceived in their daily work. Participants who had a high mean score on the questionnaire also rated themselves as more alarm fatigued (*r*=0.45 in study 1 and *r*=53 in study 2). This positive correlation indicates the convergent validity of the questionnaire. Similarly, both studies demonstrated that participants with a high mean score on the questionnaire perceived more alarms to be false in their ICU. In study 1 this association was stronger for factor 2 than for factor 1. This makes sense since a high score on factor 2 (ie, the alarm coping scale) indicates that alarms are not properly managed (eg, by means of patient-specific threshold customizations), which typically leads to more false alarms [30]. However, study 2 could not replicate this pattern. Future research should find an answer to this question: Do ICU staff with a high perceived percentage of false alarms tend to develop stronger alarm fatigue, or do staff that are more alarm fatigued tend to perceive more alarms as being false?

### Limitations

The fit indices RMSEA, CFI, and TLI have been shown to overestimate model fit when using the ULS estimator [31,32], potentially leading researchers to accept a bad-fitting model. Yet, in our case, other fit indices indicated a good model fit. As in our previous work [10], the assumption that participants can accurately reflect and express their own alarm fatigue as a percentage is likely flawed (otherwise, it would not be necessary to develop a questionnaire in the first place). However, most ICU nurses and physicians have heard of alarm fatigue, and we provided them with a brief recapitulation on the concept before having them answer the self-report item in each study. Likewise, it is also probably a flawed assumption that participants can accurately report the rate of false alarms, though Bliss et al [33] showed that participants were able to adapt their response frequencies to alarms based on to the perceived probability that an alarm was not false, thus suggesting that people might have an intuitive grasp of the rate of false alarms in their unit. All in all, we believe that asking these self-rating questions is a valuable method for assessing convergent validity when no other instrument is available.

### Conclusion

Our results from 2 independent studies underpin the construct validity of the CAFQa. All items consistently loaded onto the factors, as we proposed in a previous publication [10]. When conducting research or quality improvement projects in ICUs, clinical alarm researchers and clinicians can rely on this instrument to measure, compare, and benchmark the alarm fatigue of nurses and physicians.

## Abbreviations

CAFQa: Charité Alarm Fatigue Questionnaire
CFA: confirmatory factor analysis
CFI: comparative fit index
EFA: exploratory factor analysis
ICU: intensive care unit
MAR: missing at random
REDCap: Research Electronic Data Capture
RMSEA: root mean square error of approximation
RNI: relative noncentrality index
SRAF: self-reported alarm fatigue
SRMR: standardized root mean squared residual
TLI: Tucker-Lewis index
ULS: unweighted least-squares
